# Analysis of Treatment Outcomes and Prognosis After Concurrent Chemoradiotherapy for Locally Advanced Cervical Cancer

**DOI:** 10.3389/fonc.2022.926840

**Published:** 2022-07-06

**Authors:** Qing-he Peng, Kai Chen, Jun-yun Li, Li Chen, Wei-jun Ye

**Affiliations:** Department of Radiation Oncology, Sun Yat-sen University Cancer Center, State Key Laboratory of Oncology in South China, Collaborative Innovation Center for Cancer Medicine, Guangdong Key Laboratory of Nasopharyngeal Carcinoma Diagnosis and Therapy, Guangzhou, China

**Keywords:** locally advanced cervical cancer, concurrent chemoradiotherapy, short-term and long-term efficacies, chronic radiation proctics, prognostic factor

## Abstract

The aims of this study were to investigate the short-term and long-term efficacies and chronic radiotoxicity of concurrent chemoradiotherapy (CCRT) combined with image-guided adaptive brachytherapy (IGABT) in patients with locally advanced cervical cancer (LACC) and identify prognostic factors in this patient population. The clinical data of 204 patients with cervical cancer who completed CCRT and subsequent brachytherapy in our hospital between February 2015 and March 2017 were retrospectively analyzed. Short-term and long-term outcomes, chronic radiotoxicity, and prognostic factors were assessed. The median follow-up was 61.1 months. The short-term objective response (OR) rate was 85%. Lymph node metastasis before treatment was an independent predictor of OR (HR = 6.290, 95% CI: 2.211-17.897, p = 0.001). Fifty-two patients developed recurrence, with a median recurrence-free survival of 9.9 months (range, 2.4-52.2 months) and a post-recurrence survival of 12.1 months (range, 2.9-58.1 months). At 3 years, the cumulative incidence of overall recurrence was 26% (95% CI: 17-36). Multivariate analysis showed that Stage IIIB (HR = 2.332, 95% CI: 1.195-4.551, p = 0.013; reference, Stage IIB) and lymph node metastasis (HR = 4.462, 95% CI: 2.365-8.419, p < 0.001) were significant independent predictors of recurrence. Fifty-three patients developed chronic radiation proctitis (CRP). The incidence of severe CRP was approximately 5%, and the average rectal $D_{2}cm^{3}$ accumulation in patients with severe CRP was 73.4 Gy which is 3.9 Gy higher than that in patients without CRP (p = 0.013). At 4 years, the overall survival (OS) and disease-free survival rates were 65% and 62%, respectively, and lymph node metastasis before treatment was an independent prognostic risk factor for OS. The short-term and long-term efficacies of CCRT combined with IGABT for the treatment of LACC patients were relatively satisfactory. However, the short-term and long-term efficacies of patients with lymph node metastasis before treatment were poor. For patients with lymph node metastasis before treatment, more active individualized treatment strategies should be adopted. When designing a radiotherapy plan, it is necessary to strictly limit the rectal $D_{2}cm^{3}$ accumulation to prevent serious CRP.

## Introduction

According to the World Cancer Report 2020 released by the International Agency for Research on Cancer, cervical cancer is the most common malignant tumor of the female reproductive system worldwide, and it accounts for 3.1% and 3.4% of cancer-related morbidities and mortalities, respectively (1). According to the 2009 staging guidelines of the International Federation of Gynecology and Obstetrics (FIGO), locally advanced cervical cancer (LACC) includes Stages IB2 and IIA2-IVA cervical cancers, and the standard of care is brachytherapy after concurrent chemoradiotherapy (CCRT) (2, 3).

There have been great advancements in the treatment of cervical cancer in recent years, especially for LACC. For LACC, image-guided intensity modulated radiation therapy (IG-IMRT) and image-guided adaptive brachytherapy (IGABT) can more accurately provide a high dose to the gross tumor volume and reduce the dose to peripheral organs at risk (OARs) compared with 3-dimensional conformal radiation therapy and traditional 2-dimensional brachytherapy (4–8). In view of the significant improvement in local control and treatment-related toxicity, IG-IMRT plus IGABT combined with platinum-based concurrent chemotherapy has become the latest expert consensus for the treatment of LACCy (9–11). Many studies have reported the short-term and long-term efficacies of CCRT in LACC or its advantages over other treatment schemes, as well as prognostic factors (12–15). However, the specific radiotherapy techniques and chemotherapy schemes of these studies rarely reflect the latest expert consensus. Here, we retrospectively analyzed the clinical data of 278 cervical cancer patients who were treated with CCRT combined with subsequent brachytherapy by Professor Ye Weijun’s group in the Department of Radiotherapy, Sun Yat-sen University Cancer Center between February 2015 and March 2017. All patients underwent radiotherapy with IG-IMRT+IGABT and concurrent chemotherapy with cisplatin. The short-term and long-term efficacies and chronic radiotoxicity were assessed, and risk factors of relapse, metastasis, and survival were analyzed and summarized to provide a reference for individualized treatment of LACC.

## Materials and Methods

### General Clinical Data

The clinical data of 278 patients with cervical cancer who received CCRT and subsequent brachytherapy from Professor Ye Weijun’s group at the Department of Radiotherapy, Sun Yat-sen University Cancer Center between February 2015 and March 2017 were retrospectively analyzed. The inclusion criteria were as follows: (1) no previous major surgery and no history of pelvic radiotherapy; (2) treatment with radical CCRT and subsequent brachytherapy; (3) Stage IB2 or IIA2-IVA LACC according to the 2009 FIGO staging guidelines 2; (4) histological diagnosis of squamous cell carcinoma, adenocarcinoma, or adenosquamous cell carcinoma confirmed by pathological examination; and (5) clinical examination and specialized examination using MRI, PET-CT, and/or CT to evaluate the local and metastatic spread of the tumor. In detail, to obtain data regarding tumor size, vaginal involvement, and parametrial invasion, each patient in the lithotomy position was examined by two experienced gynecologic oncologists with vaginal inspection and bimanual vaginal and rectal palpation. At the same time, MRI was used to assist in the assessment of parametrial involvement. And MRI, PET-CT and/or CT were performed to assess regional lymph nodes. The exclusion criteria were as follows: (1) other synchronous malignant tumors; (2) serious untreated complications; and (3) loss to follow-up or incomplete records of relevant research data. There were 204 patients with cervical cancer who met the criteria, and the effective rate was 73.4%. **Table 1** provides details on the general clinical data.

**Table 1:** Clinical characteristics of patients with LACC.

Characteristics	Whole cohort (n = 204)
Age, y	57 (50 ~ 63)
Tumor diameter, cm	4.6 (3.5 ~ 5.5)
SCC value, ng/ml	6.3 (1.5 ~ 16.8)
HGB value, g/l	107 (94 ~ 116)
HRCTV D_90%_ (Gy)	102 (98 ~ 106)
Rectal $D_{2}cm^{3}$ (Gy)	71 (67 ~ 73)
Stage	
IB2	2 (1.0%)
IIA2	8 (3.9%)
IIB	84 (41.2%)
IIIA	18 (8.8%)
IIIB	92 (45.1%)
Histology	
Squamous cell carcinoma (SCC)	180 (88.2%)
Adenocarcinoma (AC)	14 (6.9%)
Adenosquamous carcinoma (ASC)	6 (2.9%)
Others	4 (2.0%)
Tumor morphology	
Exophytice type (EX-type)	165 (80.9%)
Endophytic type (ED-type)	21 (10.3%)
Ulcerative type (UC-type)	17 (8.3%)
Others	1 (0.5%)
Lymph node metastasis	
N0	134 (65.7%)
N1	51 (25.0%)
N2	19 (9.3%)

Values are presented as median (IQR) or n (%). N0 is metastasis-free, N1 is pelvic lymph node, and N2 includes 16 cases of para-aortic lymph nodes and 3 cases of inguinal lymph nodes.

### Treatment

All patients received CCRT followed by brachytherapy. Pelvic external irradiation was performed using image-guided volume-modulated arc therapy (VMAT) at a total dose of 45-50 Gy in 180-200 cGy/fraction. The primary tumor and metastatic pelvic lymph nodes were simultaneously administered 60 Gy in 240 cGy/fraction. The target volume was delineated according to the RTOG guidelines (16). In the late course of external irradiation, an Elekta high-dose-rate 192Ir afterloading unit was used to conduct CT image-guided IGABT, 7 Gy/fraction, 2 times/week, for a total of 2 weeks. The target volume was delineated according to the GEC-ESTRO guidelines (17). IGABT was administered using manual interstitial brachytherapy combined with intracavitary brachytherapy. Concurrent chemotherapy of 40 mg/m2 intravenous platinum was administered once a week for 5-7 weeks.

### Cumulative Dose to the Gross Tumor Volume and OARs

Using the quadratic linear model (α/β = 10 was adopted for gross tumor volume, and α/β = 3 was adopted for OARs), the physical dose of IGABT was converted into the bioequivalent dose of 2 Gy, and the dose received by 90% of the clinical target volume (HRCTV) of the primary tumor (D90%) and the irradiation dose received by 2 cm3 ( $D_{2}cm^{3}$ ) of the high-dose area to the OARs were calculated (18, 19). Then, the doses of IGABT and pelvic external irradiation were added to obtain the cumulative D90% to the HRCTV and the cumulative $D_{2}cm^{3}$ to the OARs.

### Follow-Up

Imaging and gynecological examination were used to assess the efficacy of treatment 3 months after treatment according to the Response Evaluation Criteria In Solid Tumors (20). Complete remission (CR) was defined as complete disappearance of the lesions and normal tumor marker levels for more than 4 weeks. Partial response (PR) was defined as a ≥30% decrease in the lesion diameter for more than 4 weeks. Stable disease (SD) was defined as a <30% decrease or a <20% increase in the lesion diameter. Progressive disease (PD) was defined as a ≥20% increase in the lesion diameter and an increase in the absolute value by ≥5 cm or the appearance of new lesions. The objective response (OR) rate was calculated as the percentage of patients achieving CR or PR. Late toxicities were judged as Grades 1-4 according to the EORTC/RTOG late effects of normal tissues guidelines (21–23). Chronic toxicity was judged and graded by three senior doctors in combination with clinical symptoms and endoscopic and imaging examination results. This study only reported the results of chronic radiation proctitis (CRP) because of its incidence and degree of impact.

Follow-up was conducted by hospital reexaminations and telephone calls. The cutoff date for follow-up was March 6, 2022. All patients were followed up regularly for more than 5 years after treatment or until death. The median follow-up time was 61.1 months (range, 3.3–80.7 months). Reexaminations were performed every 1-3 months in the 1st year, every 3 months in the 2nd to 3rd years, every 6 months in the 3rd to 5th years, and annually thereafter. Overall survival (OS) was defined as the time from the beginning of treatment to death for any reason or the last follow-up, and disease-free survival (DFS) was defined as the time from the beginning of treatment to recurrence, metastasis, or death for any reason or the last follow-up. Distant metastasis (DM)-free survival was defined as the time from the beginning of treatment to the first distant metastasis or death for any reason, and locoregional recurrence (LR)-free survival was defined as the time from the beginning of treatment to the first locoregional recurrence

### Statistical Processing

SPSS V.26.0 (IBM, New York, USA) and GraphPad Prism V.9.0 (GraphPad Software Company, Beijing, China) software were used for statistical analysis. For the univariate analysis of short-term efficacy, the Mann-Whitney U test was used for categorical data and χ^2^ test was used for continuous data; binary logistic regression analysis was used for multivariate analysis. The relationship between CRP and rectal $D_{2}cm^{3}$ was assessed by ANOVA, and LSD analysis was used for multiple comparisons. The Kaplan-Meier method was used for the analysis of recurrence and survival, and the Cox risk regression model was used for univariate and multivariate analysis. P < 0.05 indicated statistical significance.

## Results

### Analysis of Short-Term Efficacy and Influencing Factors

**Figure 1:**
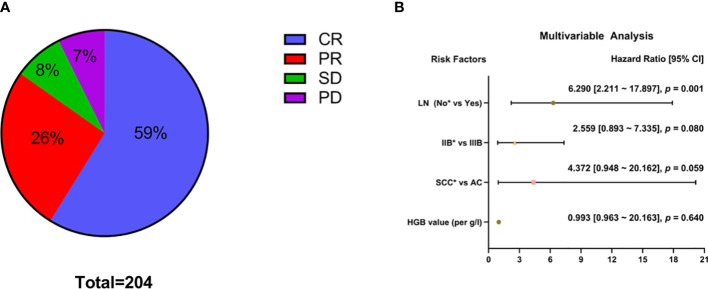
Analysis of short-term efficacy (N = 204). Specific patterns of **(A)** short-term efficacy. **(B)** Multivariable analysis; *Reference item.

Three months after treatment by CCRT combined with IGABT, 120 patients (59%) had achieved CR, 53 (26%) had achieved PR, 16 (8%) had SD, and 15 (7%) had PD (**Figure 1A**). The OR rate was 85% (173/204).

Univariate analysis showed that histological type (squamous cell carcinoma vs adenocarcinoma: χ² = 4.183, p = 0.041) and lymph node metastasis before treatment (χ² = 22.532, p < 0.001) had significant effects on short-term efficacy (**Table 2**). Factors with p < 0.2 in the univariate analysis (HGB level before treatment, histological type, FIGO stage, and lymph node metastasis before treatment) were included in the multivariate analysis. The results showed that CCRT combined with IGABT had poor short-term efficacy in LACC patients with lymph node metastasis before treatment (HR = 6.290, 95% CI: 2.211-17.897, p = 0.001), whereas HGB levels (OR = 0.993, 95% CI: 0.963-1.024, p = 0.640), histology (adenocarcinoma vs. squamous cell carcinoma: OR = 4.372, 95% CI: 0.948-20.162, p = 0.059), and tumor stage (Stage IIIB vs Stage IIB: OR = 2.559, 95% CI: 0.893-7.335, p = 0.080) were not significantly associated with short-term efficacy (**Figure 1B**).

**Table 2:** Univariate analysis of short-term efficacy in patients with LACC.

Factors	OR (N = 173)	nOR (N = 31)	*Z*/*χ²*	*p* value^#^
Age, y	57 (51 ~ 63)	54 (46 ~ 65)	-1.003	0.316
Tumor diameter, cm	4.7 (3.5 ~ 5.5)	4.0 (3.4 ~ 6.0)	-0.099	0.921
SCC value, ng/ml	6.4 (1.5 ~ 15.8)	3.8 (1.1 ~ 20.8)	-0.246	0.806
HGB value, g/l	108 (95 ~ 117)	100 (88 ~ 113)	-1.685	0.092
HRCTVTD (Gy)	88.1 (86.2 ~ 91.6)	89.7 (86.0 ~ 91.8)	-0.943	0.346
Histology			4.183^b^	**0.041**
SCC	158 (87.8%)	22 (12.2%)		
AC	9 (64.3%)	5 (35.7%)		
Tumor morphology			0.048^b^	0.826
Exophytic (EX-type)	141 (85.5%)	24 (14.5%)		
Endophytic (ED-type)	17 (81.0%)	4 (19.0%)		
Stage			3.242	0.072
IIB	75 (89.3%)	9 (10.7%)		
IIIB	73 (79.3%)	19 (20.7%)		
Lymph node metastasis			21.789	**< 0.001**
No	125 (93.3%)	9 (6.7%)		
Yes	48 (68.6%)	22 (31.4%)		

Values are presented as median (IQR) or n (%). ^b^Revised Pearson chi-square test; **^#^** Bold indicates significant differences; OR is objective responses; nOR means no remission.

### Analysis of Recurrence and Influencing Factors

**Figure 2:**
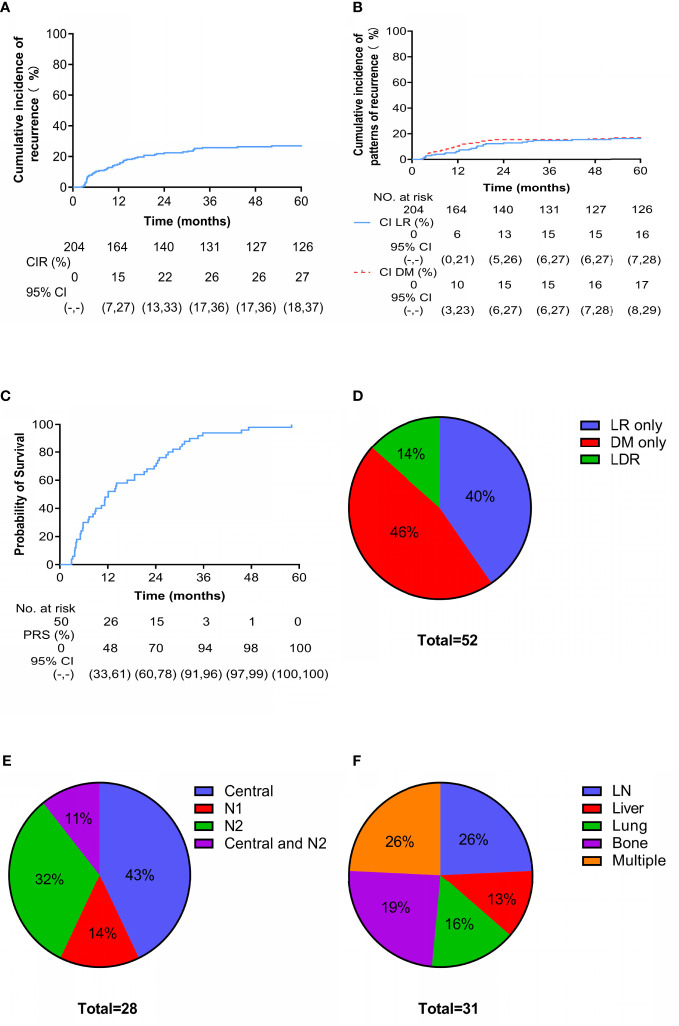
Cumulative incidence and patterns of recurrence. Cumulative incidence of **(A)** overall recurrence and **(B)** locoregional (LR) versus distant metastasis (DM) since treatment. Cumulative incidence of **(C)** death after recurrence. Site-specific patterns of **(D)** recurrence, **(E)** LR (N1 are pelvic lymph nodes and N2 are para-aortic lymph nodes or/and inguinal lymph nodes) and **(F)** DM.

During the follow-up period, 52 patients experienced recurrence (including LR and DM), with a median recurrence-free survival of 9.9 months (range, 2.4-52.2 months). At 3 years, the cumulative incidence of overall recurrence (CIR) was 26% (95% CI: 17-36) (**Figure 2A**), and the LR and DM rates were 15% (95% CI: 6-28) and 15% (95% CI: 6-27), respectively (**Figure 2B**). Fifty patients with recurrence died; the median post-recurrence survival was 12.1 months (range, 2.9-58.1 months), and more than 90% of patients with recurrence died within 3 years after recurrence (**Figure 2C**). Twenty-one (40%), 24 (46%), and 7 (14%) patients had LR only, DM only, and locoregional and distant recurrence (LDR), respectively (**Figure 2D**). Of the 28 patients with LR, 12 had central recurrence (cervical/parauterine/vaginal), 4 had pelvic lymph node metastasis (N1), 9 had paraaortic/inguinal lymph node metastasis (N2), and 3 had central type combined with N2 (**Figure 2E**). There were 31 cases of DM, and patients often had metastases at multiple sites. The most commonly affected parenchymal organs were the lungs and liver; the lymph nodes and bone were also common metastatic sites (**Figure 2F**).

**Figure 3:**
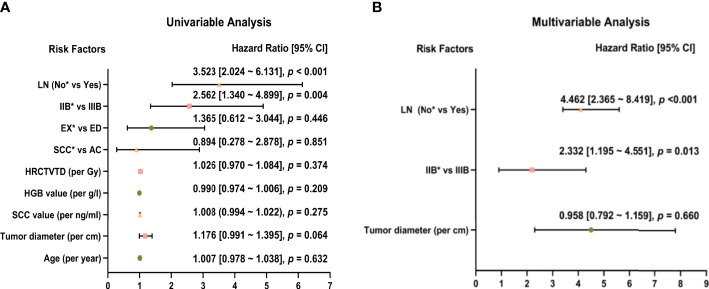
Univariate **(A)** and multivariate **(B)** analysis of 52 patients with recurrence. *Reference item.

Univariate analysis showed that stage was significantly associated with recurrence (Stage IIIB vs Stage IIB: HR = 2.562, 95% CI: 1.340-4.899, p = 0.013), and lymph node metastasis before treatment was a high-risk factor for recurrence (HR = 3.523, 95% CI: 2.024-6.131, p < 0.001). Age, tumor diameter, SCC level before treatment, HGB level before treatment, HRCTV D90%, histology, and tumor growth morphology (exophytic or endophytic) were not significantly associated with recurrence (**Figure 3A**). Factors with p < 0.2 in the univariate analysis (tumor diameter, FIGO stage, and lymph node metastasis before treatment) were included in the multivariate analysis. Tumor stage (Stage IIIB vs. Stage IIB: HR = 2.332, 95% CI: 1.195-4.551, p = 0.013) and lymph node metastasis before treatment (HR = 4.462, 95% CI: 2.365-8.419, p < 0.001) were both significant independent predictors of recurrence (**Figure 3B**).

### Relationship Between CRP and Dosimetry

**Figure 4:**
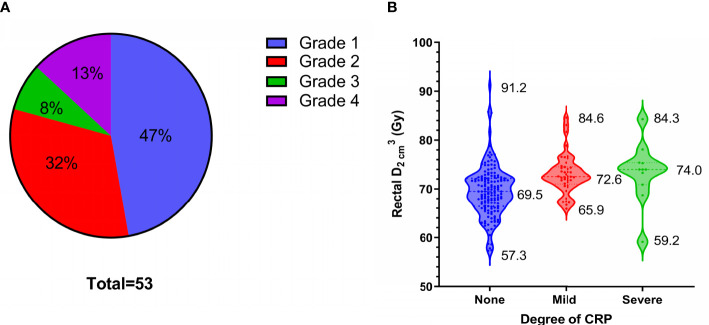
53 patients of chronic radiation proctitis (CRP). **(A)** The proportion of each grade of CRP. **(B)** Rectal $D_{2}cm^{3}$ grouped by degree of CRP.

Fifty-three patients (26%) developed CRP, including 25, 17, 4, and 7 with Grades 1, 2, 3, and 4 CRP, respectively (**Figure 4A**). Grades 1 and 2 CRP were classified as mild (42 cases), and Grades 3 and 4 CRP were classified as severe (11 cases). The incidence of severe CRP was approximately 5%. The average cumulative rectal $D_{2}cm^{3}$ of patients without CRP, those with mild CRP, and those with severe CRP were 69.5 Gy ( ± 5.1 Gy, range, 57.3-91.2 Gy), 73.1 Gy ( ± 4.0 Gy, range, 65.9-84.6 Gy), and 73.4 Gy ( ± 6.2 Gy, range, 59.2-84.3 Gy), respectively. The cumulative rectal $D_{2}cm^{3}$ of patients without CRP was 3.6 Gy lower than that of patients with mild CRP (p < 0.001) and 3.9 Gy lower than that of patients with severe CRP (p = 0.013). There was no statistically significant difference in the cumulative rectal $D_{2}cm^{3}$ between patients with mild and severe CRP (p = 0.853) (**Figure 4B** and **Table 3**).

**Table 3:** Relationship between degree of CRP and $D_{2}cm^{3}$ of the rectum.

Organ	Degree of CRP			*F*	*p* value^#^
Rectum	None (n = 151)	Mild (n = 42)	Severe (n = 11)		
$D_{2}cm^{3}$ (Gy)	69.5 ± 5.1	73.1 ± 4.0	73.4 ± 6.2	10.598	**<0.001**
$D_{2}cm^{3}$ (Gy)	**Pairwise comparison**		**Mean value**	**Difference value**	***P* value^#^**
	None vs Mild		69.5 vs. 73.1	-3.6	**<0.001**
	None vs Severe		69.5 vs. 73.4	-3.9	**0.013**
	Mild vs Severe		73.1 vs. 73.4	-0.3	0.853

^#^Bold indicates significant differences.

### Analysis of Recurrence and Influencing Factors

**Figure 5:**
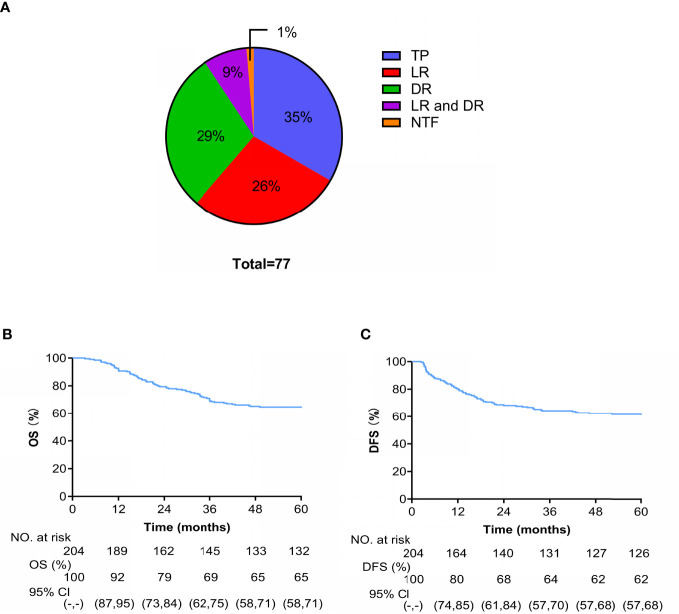
**(A)** Cause-specific of death (TP is tumor progression and NTF is non-tumor factors). Kaplan-Meier curves of OS **(B)** and **(C)** DFS.

A total of 77 patients died during the follow-up: 1 patient died of acute leukemia, 27 (35%) died of tumor progression, 20 (26%) died of LR, 23 (29%) died of DM, and 7 (9%) died of LDR (**Figure 5A**). At 4 years, the cumulative OS and DFS rates were 65% and 62%, respectively (**Figures 5B, C**).

Univariate analysis showed that tumor diameter, HGB level before treatment, FIGO stage, and lymph node metastasis before treatment were significantly associated with OS. Factors with p < 0.2 in the univariate analysis (tumor diameter, SCC and HGB levels before treatment, FIGO stage, and lymph node metastasis before treatment) were included in the multivariate analysis. Lymph node metastasis before treatment was the only independent prognostic risk factor for OS (HR = 6.765, 95% CI: 3.826-11.964, p < 0.001) (**Table 4**).

**Table 4:** Risk factors of OS identified by univariable and multivariable analysis.

Variable	Univariable HR (95% CI)	*p* value^#^	Multivariable HR (95% CI)	*p* value^#^
Age, per year	1.006 (0.980 ~ 1.031)	0.666		
Tumor diameter, per cm	1.242 (1.080 ~ 1.429)	**0.002**	0.948 (0.806 ~ 1.115)	0.518
SCC value, per ng/ml	1.011 (1.000 ~ 1.022)	0.057	1.012 (0.998 ~ 1.027)	0.101
HGB value, per g/l	0.984 (0.971 ~ 0.997)	**0.015**	0.988 (0.973 ~ 1.003)	0.116
HRCTV D_90%_ (per Gy)	1.011 (0.965 ~ 1.059)	0.648		
SCC^*^ vs AC	1.393 (0.603 ~ 3.218)	0.437		
EX-type^*^ vs ED-type	1.497 (0.765 ~ 2.929)	0.239		
IIB^*^ vs IIIB	1.826 (1.093 ~ 3.051)	**0.022**	1.500 (0.884 ~ 2.547)	0.133
LN (No^*^ vs Yes)	5.866 (3.642 ~ 9.449)	**< 0.001**	6.765 (3.826 ~ 11.964)	**< 0.001**

^*^ Reference item; **^#^** Bold indicates significant differences.

## Discussion

In this study, patients with LACC received concurrent VMAT external irradiation and cisplatin-based chemotherapy combined with IGABT, and the OR and OS rates were 85% and 65%, respectively. Previous studies reported that, under the standard treatment scheme, the local control and long-term OS rates of LACC were approximately 80% and 60%, respectively (24–26). Our results were similar to those in these studies, indicating that our efficacy was satisfactory. The 5-year CIR was 27%, among which LR only, DR only, and LDR accounted for 40%, 46%, and 14% of recurrence patterns, respectively. In similar studies, the recurrence rate of LACC patients was 20%-30% (27, 28). The most common recurrence pattern in these studies was DR with lymph node metastasis, which is similar to our results.

CRP is a common complication of radiotherapy for cervical cancer (29, 30). Patients who develop CRP may have permanent changes in defecation habits, and symptoms such as gastrointestinal bleeding may also occur, which seriously affects patient quality of life. In this study, the incidences of mild CRP (Grades 1-2) and severe CRP (Grades 3-4) were approximately 21% and 5%, respectively. Dosimetry analysis showed that the average rectal cumulative $D_{2}cm^{3}$ of patients with severe CRP was 73.4 Gy, which was 3.9 Gy higher than that of patients without CRP. Therefore, rectal $D_{2}cm^{3}$ should be fully considered and limited when planning radiotherapy.

We analyzed factors influencing short-term and long-term outcomes, including clinical features and radiation doses. There were many single factors affecting prognosis, but local lymph node metastasis before treatment was the main independent risk factor of short-term and long-term outcomes. Previous studies have reported that pelvic or abdominal paraaortic lymph node metastasis before treatment is an independent prognostic risk factor for LACC patients (31–33). In addition, the size and number of metastatic lymph nodes are independent prognostic factors for LACC (34, 35). We did not find any influence of the HRCTV $D_{2}cm^{3}$ on short-term or long-term outcomes, which might be because the HRCTV $D_{2}cm^{3}$ was over 83 Gy in all patients, over 85.0 Gy in 97% of patients, and over 90 Gy in 91% of patients. Bandyopadhyay et al. (36) reported a significant difference in the HRCTV $D_{2}cm^{3}$ between patients who did and did not develop recurrence (83.97 Gy and 77.96 Gy, respectively) among patients with LACC treated with CCRT combined with IGABT. Therefore, we suggest that the cumulative HRCTV $D_{2}cm^{3}$ of radiotherapy for LACC patients should be above 85 Gy.

The limitation of this study is its retrospective design. Further, although the number of cases in this study was relatively large, we included mainly patients with FIGO Stage IIB and IIIB disease, and there were no patients with Stage IVA disease. Additionally, most patients had squamous cell carcinoma, and the specific locations and numbers of pelvic metastatic lymph nodes were not statistically analyzed before treatment. Many studies have reported that patients with cervical squamous cell carcinoma have better short-term efficacy, OS, and DFS than those with adenocarcinoma (37–39), which is speculated to be related to the low radiosensitivity of adenocarcinoma. Tumor diameter has been also reported as an independent prognostic factor for LACC (40), which is considered to be related to the proportion of hypoxic tumor cells, as hypoxic cells are less sensitive to radiation. Furthermore, we assessed pre-treatment metastatic lymph nodes by MRI, PET-CT, and/or CT, which is sometimes difficult and may lead to some errors. A systematic review covering two hundred and twenty studies concluded that minimally invasive laparoscopic staging in patients for LACC is feasible, especially in patients with microscopic metastasis. What’s more, overtreatment could be spared by the strategy (41). Objectively, the minimally invasive laparoscopic staging indeed has some advantages over imaging examination, but superior skills and constant training are required staging procedure to avoid operative complications. In addition, we would take further aggressive treatment such as surgery, re-radiotherapy and re-chemotherapy for some recurrent patients. Nevertheless, in order to ensure the consistency of treatment scheme of all patients enrolled in the study, we excluded patients who accepted aggressive treatment after recurrence. Some studies have reported the use of expanded lateral pelvic resection and therapeutic lymphatic dissection for gynecological malignancies (42, 43). This strategy may be a good option for recurrent or chemoradiotherapy-insensitive LACC.

In summary, we examined the short-term and long-term outcomes after concu**r**rent VMAT external radiotherapy and platinum-based chemotherapy combined with IGABT in patients with LACC. The OR rate was 85%, the cumulative OS and DFS rates at 5 years were 65% and 62%, respectively, and the short-term and long-term efficacies were satisfactory. Lymph node metastasis before treatment was an independent risk factor of short-term and long-term outcomes. The incidence of severe CRP was 5%, and the average cumulative rectal $D_{2}cm^{3}$ in patients with severe CRP was 73.4 Gy, which was 3.9 Gy higher than that in patients without CRP. For patients with lymph node metastasis before treatment, more active individualized treatment measures should be taken. When designing a radiotherapy plan, it is necessary to strictly limit the rectal $D_{2}cm^{3}$ accumulation to prevent serious CRP.

## Data Availability Statement

The datasets of this research are also backed up on the Research Data Deposit (RDD, https://www.researchdata.org.cn, approval number: RDDA2022129802) and are available on reasonable request.

## Ethics Statement

The studies involving human participants were reviewed and approved by Sun Yat-sen University Cancer Center. The patients/participants provided their written informed consent to participate in this study.

## Author Contributions

Q-hP is responsible for project design and article writing, KC and J-yL are responsible for patient diagnosis and treatment, LC guides the project, and W-jY is in charge of clinical guidance and article revision. All authors contributed to the article and approved the submitted version.

## Funding

This work was supported by the National Natural Science Foundation of China (12075329)

## Conflict of Interest

The authors declare that the research was conducted in the absence of any commercial or financial relationships that could be construed as a potential conflict of interest.

## Publisher’s Note

All claims expressed in this article are solely those of the authors and do not necessarily represent those of their affiliated organizations, or those of the publisher, the editors and the reviewers. Any product that may be evaluated in this article, or claim that may be made by its manufacturer, is not guaranteed or endorsed by the publisher.
